# Decoding the impact of environmental shifts on snail density dynamics in the Yangtze River basin: a 26-year study

**DOI:** 10.1186/s13071-025-06782-3

**Published:** 2025-04-26

**Authors:** Yanfeng Gong, Shiqing Zhang, Dandan Lin, Yu Cai, Shangbiao Lv, Mao Zheng, Benjiao Hu, Xiaolan Lei, Ning Xu, Jiamin Wang, Junhui Huang, Yu Zhou, Liyun Zhu, Yue Chen, Qingwu Jiang, Shizhu Li, Yibiao Zhou

**Affiliations:** 1 Fudan University School of Public Health, Building 8, 130 Dong’an Road, Xuhui District, Shanghai, 200032 China; 2 Key Laboratory of Public Health Safety, Ministry of Education, Fudan University, Building 8, 130 Dong’an Road, Xuhui District, Shanghai, 200032 China; 3 Fudan University Center for Tropical Disease Research, Building 8, 130 Dong’an Road, Xuhui District, Shanghai, 200032 China; 4 Department of Schistosomiasis Control and Prevention, Anhui Institute of Parasitic Diseases, Hefei, 230061 China; 5 Jiangxi Provincial Institute of Parasitic Diseases, Nanchang, 330096 China; 6 Hunan Institute for Schistosomiasis Control, Jin’e Middle Road, Yueyang, 414021 Hunan China; 7 School of Epidemiology and Public Health, Faculty of Medicine, University of Ottawa, 600 Peter Morand Crescent, Ottawa, ON K1G 5Z3 Canada; 8 National Key Laboratory of Intelligent Tracking and Forecasting for Infectious Diseases, National Institute of Parasitic Diseases at Chinese Center for Disease Control and Prevention (Chinese Center for Tropical Diseases Research), Shanghai, 200025 China

**Keywords:** *Oncomelania hupensis* snail, Schistosomiasis, Flooding duration, Bayesian model, Surveillance

## Abstract

**Background:**

With the intensification of climate change and human engineering activities, environmental changes have affected schistosome-transmitting snails. This study explored the influence of environmental changes on the evolution of snail populations.

**Methods:**

Data from annual snail surveys and related factors such as hydrology, temperature, vegetation, etc., on nine bottomlands from 1997 to 2022 were collected retrospectively from multiple sources. Interpretable machine learning and the Bayesian spatial-temporal model assessed the relationship between environmental change and snail density.

**Results:**

Between 1997 and 2003, mean snail density was in a high-level fluctuation stage. From 2003 to 2012, it declined significantly from 0.773/0.1 m^2^ to 0.093/0.1 m^2^. However, it increased by 27.6% between 2013 (0.098/0.1 m^2^) and 2022 (0.125/0.1 m^2^). Since operation of the Three Gorges Dam (TGD) began in 2003, the duration of bottomland flooding decreased from 122 days (1997–2003) to 57 days (2003–2012) and then rebounded in 2012–2022, which was noticeable in the Anhui Section. The ground surface temperature and night light index of the bottomlands increased from 1997 to 2022. After adjusting for confounding factors (e.g. rainfall, temperature, and vegetation), the relative risk (RR) of increased snail density rose with flooding duration of between 20 and 100 days but decreased with flooding duration of > 100 days. Snail density showed an “L”-shaped relationship with the night light index, and the RR of increased snail density was lower at a higher night light index. Compared with bottomlands in the first quartile cluster of ground surface temperature, bottomlands in the second, third, and fourth quartile clusters of ground surface temperature had higher snail density RR values of 1.271 (95% *CI* 1.082–1.493), 1.302 (95% *CI* 1.146–1.480), and 1.278 (1.048, 1.559), respectively.

**Conclusions:**

The TGD lowered the water level and flooding duration, which were not conducive to snail population growth. However, over time, the inhibitory effect of the TGD on snails may have been weakening, especially in areas far from the TGD. In recent years, the rebound of snail density may have been related to the rise in water levels and the change in the microenvironment. Establishing an efficient monitoring and response system is crucial for precisely controlling snails.

**Graphical Abstract:**

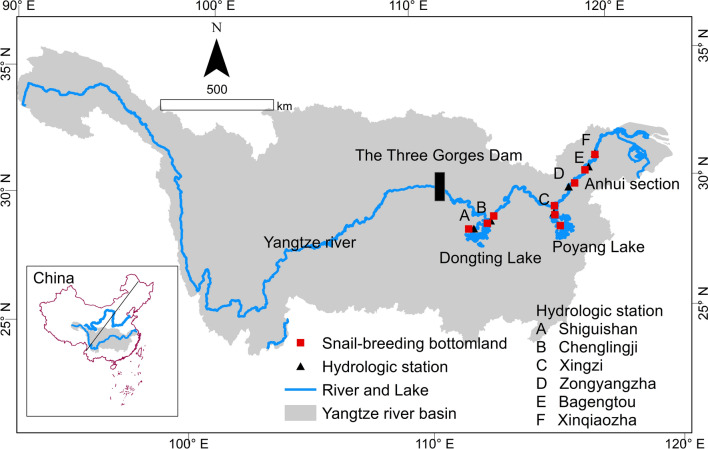

**Supplementary Information:**

The online version contains supplementary material available at 10.1186/s13071-025-06782-3.

## Background

China has significantly reduced schistosomiasis infections by implementing flexible control strategies at different times and is currently promoting the elimination of schistosomiasis [1, 2]. However, as a natural epidemic disease, the risk of schistosomiasis transmission has existed for a long time because the natural and social environment in the epidemic area has not undergone fundamental changes [3]. The *Oncomelania hupensis* snail is the only intermediate host involved in the transmission of *Schistosoma japonicum* [1, 3]. Snail control is an effective measure recommended by WHO to eliminate the hidden dangers of schistosomiasis [4]. However, the area of snail habitats has remained at a high level of 3.6 billion m^2^ since 2000, with emerging habitats continuing to appear [5].

The dynamics of the snail life cycle are influenced by both natural environmental and human activities. Natural factors mainly include climate change and major flood events, while human activity involves water conservancy projects (e.g. dam construction) [6, 7]. Temperature and precipitation are key limiting factors regarding snail distribution. Studies by Zhou et al. [8] suggest that with global warming, snail habitats may expand northward into the Yangtze River basin, reaching northern Jiangsu and Anhui. Following the 1998 Yangtze River flood event, the areas of re-emerging and newly established snail habitats in the basin were 2.6 and 2.7 times larger, respectively, than in years with normal hydrological conditions [9]. Water conservancy projects, such as dams and irrigation systems, often alter water distribution and create favorable habitats for snails [10, 11]. In the Senegal River basin, evidence suggests that after the construction of the Diama Dam, ecological changes promoted the growth and reproduction of freshwater snails [12]. The proportion of schistosome-transmitting snails among collected specimens increased from 22 to 60% [12]. This chain reaction—dam construction, environmental change, and proliferation of freshwater snails—has drawn significant attention [10–12].

The Three Gorges Dam (TGD) is a large-scale hydroelectric power project built in the upper reaches of the Yangtze River and has been in operation since 2003 [13]. Due to the artificial regulation of water resources by the TGD, the downstream water regime has undergone significant changes [13, 14]. Li et al. [15] showed that following the TGD, the ecological environment (such as volume of runoff, sediment volume, and sedimentation rate) has changed in Dongting Lake, causing the occurrence rate of frames with living snails and living snail density to decrease by > 80% from 2003 to 2015. Wu et al. [16] showed that the TGD reduced the risk of flooding and living snail density. Fundamental changes in water environment inevitably impact various aspects such as the microenvironment (vegetation, sunshine light, and temperature) and utilization of the bottomland. These ecological elements are also important components of the survival conditions required for snails, but research on the impact of the water environment and related factors on snail distribution are still inadequate.

Artificial intelligence (AI) and new model algorithms play a significant role in monitoring snail habitats and exploring shifts in snail density in their environment [17]. First, AI can process remote sensing data from satellites, drones, and other geospatial sources in real time to track key environmental variables such as water body, temperature, and land cover [18]. Second, new model algorithms can detect spatial and temporal patterns that may not be apparent using traditional analysis. For instance, Qureshi et al. [19] used machine learning and SHapley Additive exPlanations (SHAP) to analyze the flight trajectory characteristics of mosquitoes on mosquito nets and provide direction for mosquito interventions. Third, AI-driven automated monitoring systems can analyze real-time data from environmental sensors, field surveys, and image recognition techniques to detect changes in disease vectors. Explainable machine learning and Bayesian models are increasingly applied in vector-borne disease analysis because of their flexibility and efficiency in handling complex data. For instance, Villela et al. [20] constructed a Bayesian hierarchical model to estimate the abundance and spatial density of *Aedes aegypti*, demonstrating the potential of related models in assessing vector populations and their environmental dynamics.

This study aimed to explore the long-term evolutionary characteristics of snail density related to the construction of TGD using explainable machine learning and a Bayesian spatial-temporal model by collecting continuous snail survey data from nine bottomlands from 1997 to 2022. This study could provide a reference for understanding the evolution of snails associated with environmental change and ecological snail control.

## Methods

### Study area

The Yangtze River basin is characterized by a humid subtropical climate with distinct monsoon influences [21]. Precipitation increases from southwest to northeast across the basin, with annual rainfall ranging from 800 to 2000 mm. The region has small temperature variations and high humidity, with an average annual temperature of 15 °C to 19 °C. The topography is primarily flat, consisting of plains and low hills, interspersed with numerous rivers, lakes, and wetlands. The region is influenced by both monsoon and tropical climate systems, leading to significant water level fluctuations and frequent flooding [22]. These conditions support the basin's rich vegetation and biodiversity, including snail populations.

**Fig. 1:**
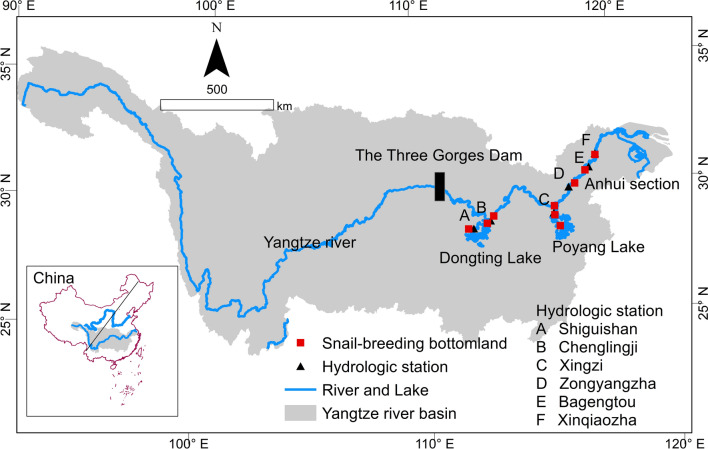
The study bottomlands in the Yangtze River basin

The Yangtze River, spanning a colossal 6300 km and draining an expansive 1.8 million km^2^ from the Tibetan Plateau to eastern China, is the largest river in Asia. Situated in the upper reaches of the Yangtze River, the TGD began filling with water in June 2003 [13]. In this study, we focused on the downstream region of the TGD, selecting nine snail-infested sites from Dongting Lake, Poyang Lake, and the Anhui section (Fig. 1). In each region, three study sites were chosen, representing high-, medium-, and low-elevation bottomlands (Additional file 1: Table S1).

### Data collection and preprocessing

The snail survey was conducted annually in the nine bottomlands each spring from 1997 to 2022 (Fig. 1; Additional file 1: Table S1). This was performed by using the Chinese traditional method of systematic sampling (0.1 m^2^-sized frames) [23, 24]. All snails within the square frames were collected and brought to the laboratory. These collected snails were then counted and crushed to determine their vitality [23, 24]. The overall mean density of living snails and living snail density at different elevations were obtained by dividing the number of living snails by the number of snail survey frames.

Six hydrological stations in proximity to the study bottomland within the Dongting Lake area, Poyang Lake area, and Anhui section of the Yangtze River were selected. From 1997 to 2022, daily water level data at 8 a.m. were retrospectively collected from these hydrological stations. The average elevation of each bottomland was derived from elevation grid data (https://www.resdc.cn/). A bottomland was classified as submerged if the difference between the daily water level at the hydrological station and the average elevation of the beach was > 0 [25]. The flooding duration (FD; unit: days) was defined as the number of days that a bottomland was submerged in water during a year.

Vegetation is an important factor affecting snails, which can usually regulate the temperature and humidity of snail habitat and serve as their food. The normalized difference vegetation index (NDVI) was an accurate gauge of vegetation greenness, photosynthetic intensity, metabolic activity, and seasonal and interannual variations. This study employed the maximum composites method [26] on monthly NDVI grid data from the PKU GIMMS NDVI [27], enabling the derivation of annual NDVI grid data between 1997 and 2022.

The nighttime light conditions of the bottomland and its surroundings can reflect the development and utilization of these areas, as well as the infrastructure construction, which indirectly affects the breeding and reproduction of snails [28]. The nighttime light index (NL) used in this study is derived from the corrected DMSP-OLS-like data for mainland China (continuously updated since 1992), which were obtained by integrating DMSP-OLS and SNPP-VIIRS data [29]. The data were sourced from the National Earth System Science Data Center (https://www.geodata.cn/data/).

The sunshine hour (SSH/hour), ground surface temperature (GST/℃), precipitation (Pre/mm), air temperature (Tem/℃), and relative humidity (RH/%) were acquired from the National Meteorological Data Service Platform (http://cdc.cma.gov.cn/). Kriging interpolation techniques were applied to generate raster data [30]. Subsequently, the data for the nine bottomlands were extracted from this interpolated dataset. Extreme temperature conditions were gauged using the average minimum temperature in January (Tmin/℃) and the average maximum temperature in July (Tmax/℃), sourced from the National Earth System Science Data Center (http://www.geodata.cn/).

There may be correlations between the above variables, which may cause collinearity and modeling errors, so we conducted a correlation analysis to exclude variables with high correlations. The Spearman correlation coefficients between the other variables were all < 0.5, with only those between GST and Tem, as well as FD and RH, > 0.7 (Additional file 1: Table S2). To avoid collinearity, Tem and RH were excluded from the analysis. The final variables included in the analysis were FD, NDVI, NL, Tmin, GST, Pre, SSH, and Tmax.

### Statistical analysis

Previous theoretical studies have suggested potential differences in water and sediment dynamics during various operational stages of the TGD [31]. Therefore, this study divided the research time into pre-TGD (1997–2002) and post-TGD (the 2003–2012 and 2013–2022 periods). In addition, microenvironments such as temperature and sunshine may also change over time. The Shapiro-Wilk normality test was employed to assess the distribution of environmental factors, which revealed a departure from the normal distribution. The *P*-values from the Shapiro-Wilk test for all variables were < 0.05 (Additional file 1: Table S3), indicating that the data did not follow a normal distribution. Therefore, non-parametric (Kruskal-Wallis test) methods were used to compare the environmental factors among different stages. Bonferroni correction was applied to mitigate the risk of Type I errors associated with multiple comparisons [32].

This study used the light gradient boosting machine (LightGBM) model with the SHAP method to discern the principal drivers influencing vegetation and amphibious freshwater snails, elucidating their relationships with core ecological factors. LightGBM continuously refines the model by fitting residuals from the current learner and employs the forward distribution algorithm for iterative training [33]. The SHAP method, a model explanation tool rooted in Shapley values, functions as a post hoc explainability scheme [34]. It elucidates the significance of each feature and the direction in which each feature influences the decision-making process of the model [35].

Although SHAP values identified the relationship between individual environmental factors and snail density, these relationships might be confounded by multiple factors, and no statistical tests have been conducted. We built a multi-factor Bayesian space-time model to further examine the snail density and related factors. The general form of the model is given as follows [36, 37]:

$${y}_{it}\sim NB\left({\pi }_{it},\text{r}\right)$$

$${\pi }_{it}=r/(r+{u}_{it})$$

$$log\left({u}_{it}\right)=log{e}_{it}+\sum_{k=1}^{k}{\beta }_{k}{X}_{itk}+\sum_{q=1}^{q}S({X}_{itq})+{u}_{i}+{v}_{i}+{\varphi }_{t}+{\gamma }_{t}+{\delta }_{it}$$

In this model, ${y}_{it}$​ represents the observed number of snails, and ${e}_{it}$ denotes the expected number of snails. The model assumes that ${y}_{it}$​ follows a negative binomial distribution with a mean ${u}_{it}={e}_{it}{\theta }_{it}$​, where ${\theta }_{it}$ represents the relative risk (RR) at site $i$ in year $t$. The parameter $r$ accounts for dispersion. ${\beta }_{k}$​ is a vector of regression parameters, and ${X}_{itk}$ represents the covariate. $S({X}_{itq})$ denotes the smoothing spline function terms used to approximate the nonlinear association between environmental variables and snail density. The terms ${u}_{i}$ and ${\varphi }_{t}$ represent the spatially unstructured and temporally structured effects, respectively; both are assume to follow a normal distribution. The spatially structured effect ${v}_{i}$ was modeled using a conditional autoregressive (CAR) process, with the CAR adjacency matrix $\text{w}$ defined by the K nearest neighbors. The snail densities in the same region were similar, so K was set to 2 in this study. The temporally structured component ${\gamma }_{t}$ was modeled using a first-order autoregression. The spatiotemporal interaction effect ${\delta }_{it}$​ was also assumed to follow a normal distribution. Hyperpriors for the precisions of the random effects were assigned as follows: ${\tau }_{u}$, ${\tau }_{\text{v}}$, ${\tau }_{\varphi }$, ${\tau }_{\gamma }$, ${\tau }_{\delta }$. Their prior distributions were ${\tau }_{u}\sim (\text{1,0.0005})$, ${\tau }_{\text{v}}\sim (\text{1,0.0005})$, ${\tau }_{\varphi }\sim (1, 0.00005)$, ${\tau }_{\gamma }\sim (\text{1,0.00005})$, ${\tau }_{\delta }\sim (\text{1,0.00005})$.

The models were fitted using the Integrated Nested Laplace Approximation method and evaluated based on the deviance information criterion (DIC). The model with the lowest DIC score was chosen as the final model for this study. The analyses were conducted in R 4.4.1 (R core team, Vienna, Austria) and ArcGIS 10.2 (Esri, Redlands, CA, USA). We considered a two-sided *P*-value < 0.05 as statistically significant.

## Results

### The characteristics of snail density

**Fig. 2:**
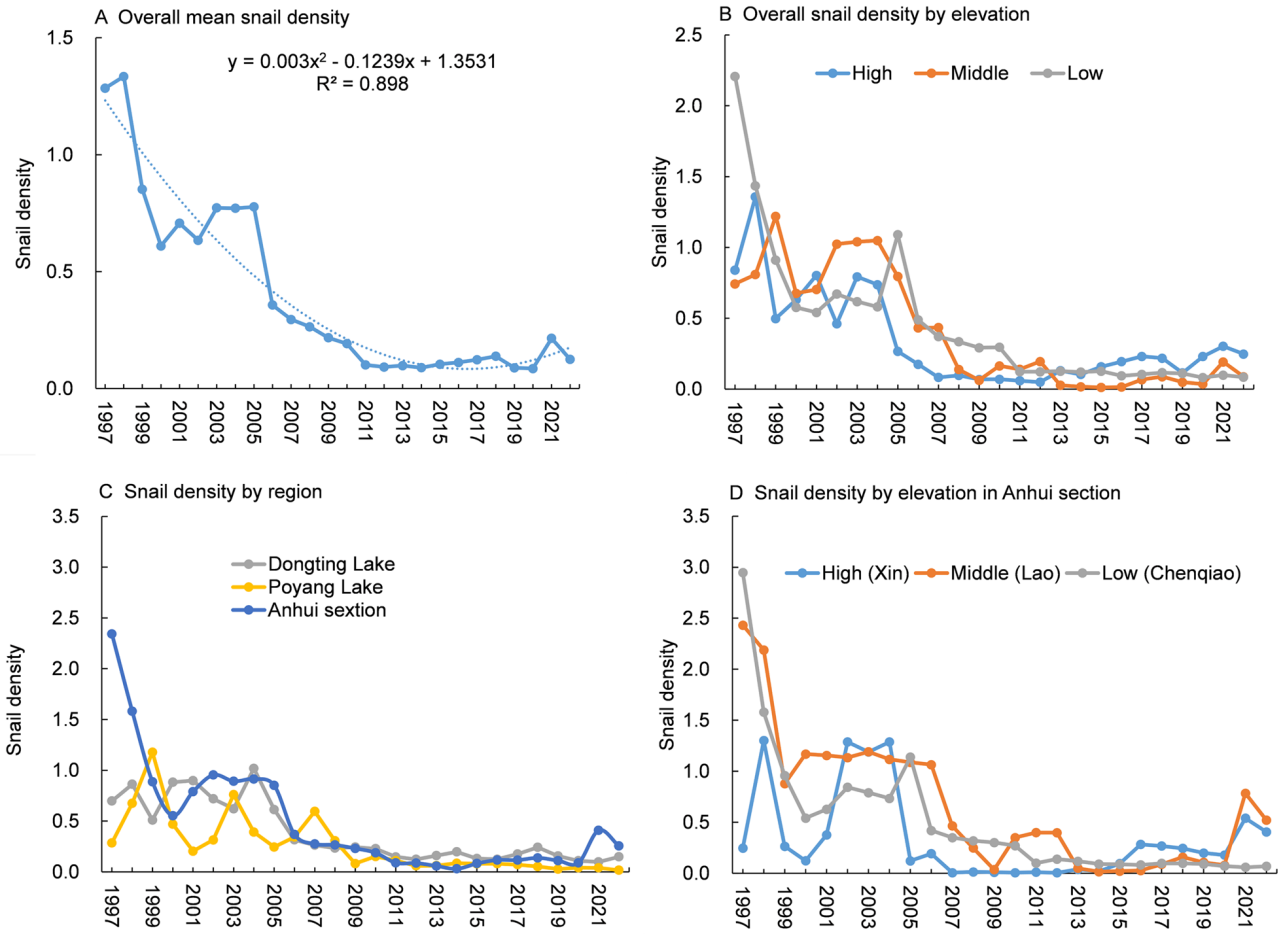
The trends in snail density from 1997 to 2022: **A** average snail density downstream from bottomlands of the dam, **B** trends in snail density at high-, medium-, and low-elevation bottomlands, **C** trends in snail density in the Dongting Lake area, Poyang Lake area, and Anhui section, **D** trends in snail density at high-, medium-, and low-elevation bottomlands in the Anhui section

Between 1997 and 2003, the snail density was in a high-level fluctuation stage, and the overall snail density dropped from 1.357 in 1998 to 0.610 in 2000 and then rebounded to 0.773 in 2003 (Fig. 2A). During the 1st decade operation of the TGD (2003–2012), the snail density in high-elevatione bottomlands began to decrease in 2003, and the snail density in medium-elevation bottomlands started to decrease in 2004 (Fig. 2B). The snail density in low-elevation bottomlands increased from 2003 to 2005 and then began to decrease sharply in 2006. However, during the 2nd decade of TGD (2013–2022), snail density experienced a rebound from 0.098/0.11 m^2^ in 2013 to 0.125/0.11 m^2^ in 2022. Notably, over half (six of nine) of the bottomlands witnessed an increase in snail density between 2013 and 2022, namely Matian, Xingang, and Junshan Park in Dongting Lake, Ganyu in Poyang Lake, and Xin and Lao in the Anhui section. The snail density in the Anhui section of the Yangtze River, which is far away from the dam, increased significantly from 0.059/0.1 m^2^ to 0.256/0.1 m^2^ (Fig. 2C, D).

### Changes in the water regime, vegetation, and microclimate

The average FD in the nine downstream bottomlands of the TGD was 73.56 ± 44.31 days for the range of years covered in this study, with the bottomlands experiencing a minimum of 5 days of flooding and a maximum of 161 days of flooding. The maximum value of annual highest water level was 41.80 m (occurring before the TGD at Shiguishan in Dongting Lake), and the minimum value of the annual lowest water level was 1.40 m (occurring after the TGD at Xinqiaozhao in Anhui section). The NDVI and NL range was from 0.31 to 0.83 and 69.31 to 256.83 for nin3 bottomlands, with the lowest values of NDVI and NL occurring before the operation of the dam. The range of RH and SSD was 76.25–84.38% and 1730–2058 h (Additional file 1: Table S4).

**Fig. 3:**
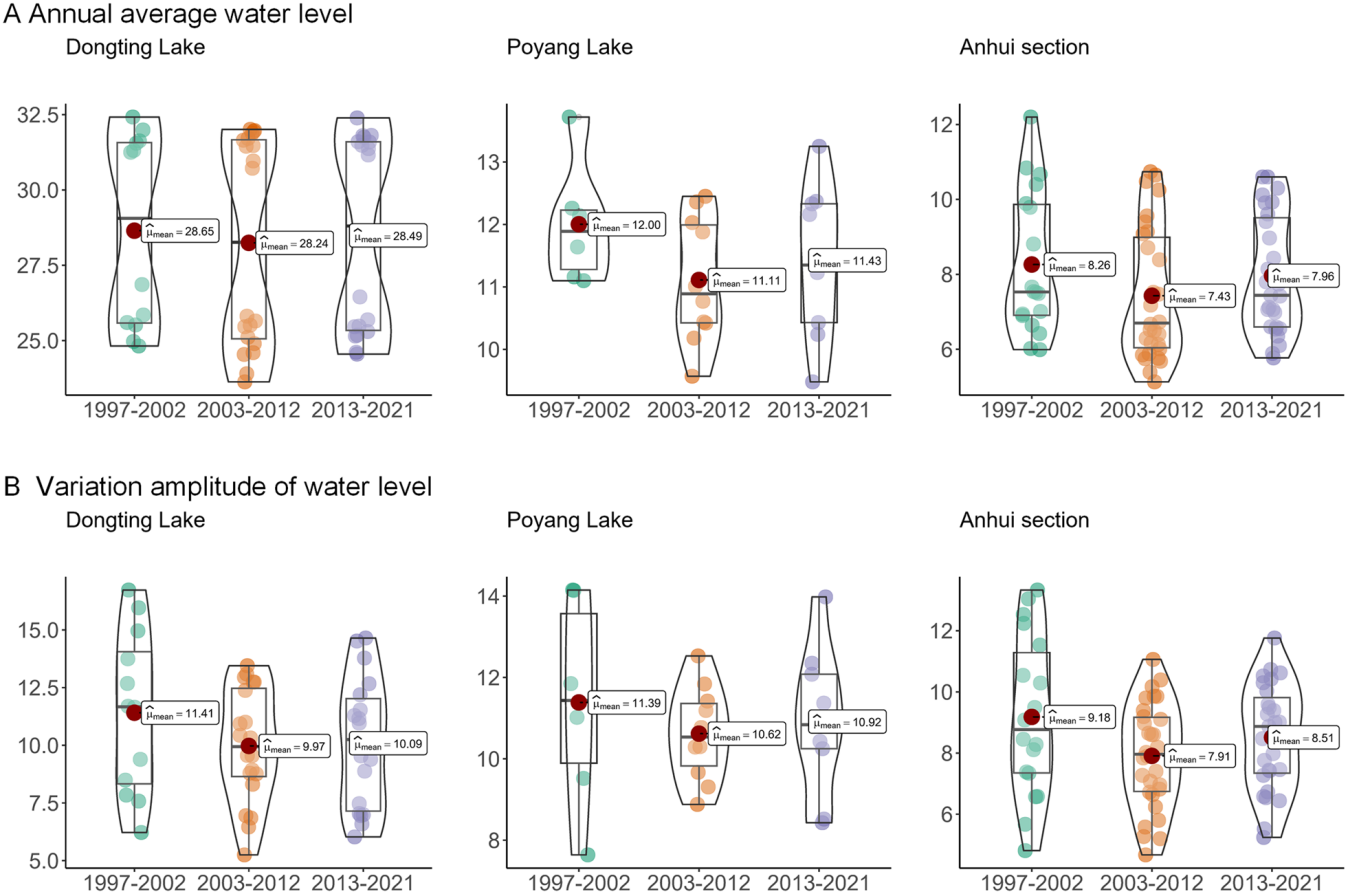
Changes in annual average water level (**A**) and variation amplitudes of water levels (**B**) during the 1997–2002, 2003–2012, and 2013–2022 periods

Following the 1st decade of the TGD between 2003 and 2012, there was a noticeable decline in both water levels and their fluctuations, as depicted in Fig. 3. However, during the 2nd decade of the TGD (2013–2022), there was a slight resurgence in water levels and their fluctuations. During 2013–2022, the annual average water levels in the Dongting Lake region, Poyang Lake region, and Anhui section of the Yangtze River rose by 0.25 m, 0.32 m, and 0.53 m, respectively, with the rise becoming more pronounced far away from the TGD. A more detailed analysis reveals that during the periods 1997–2002, 2003–2012, and 2013–2022, the average water levels in the Dongting Lake area, Poyang Lake area, and Anhui section exhibited variations of 28.65–28.24–28.49, 12.00–11.11–11.43, and 8.26–7.43–7.96, respectively. Similarly, the annual fluctuations in water levels during these periods in the Dongting Lake area, Poyang Lake area, and Anhui section showed changes of 11.41–9.97–10.09, 11.39–10.62–10.92, and 9.18–7.91–8.51, respectively.

**Fig. 4:**
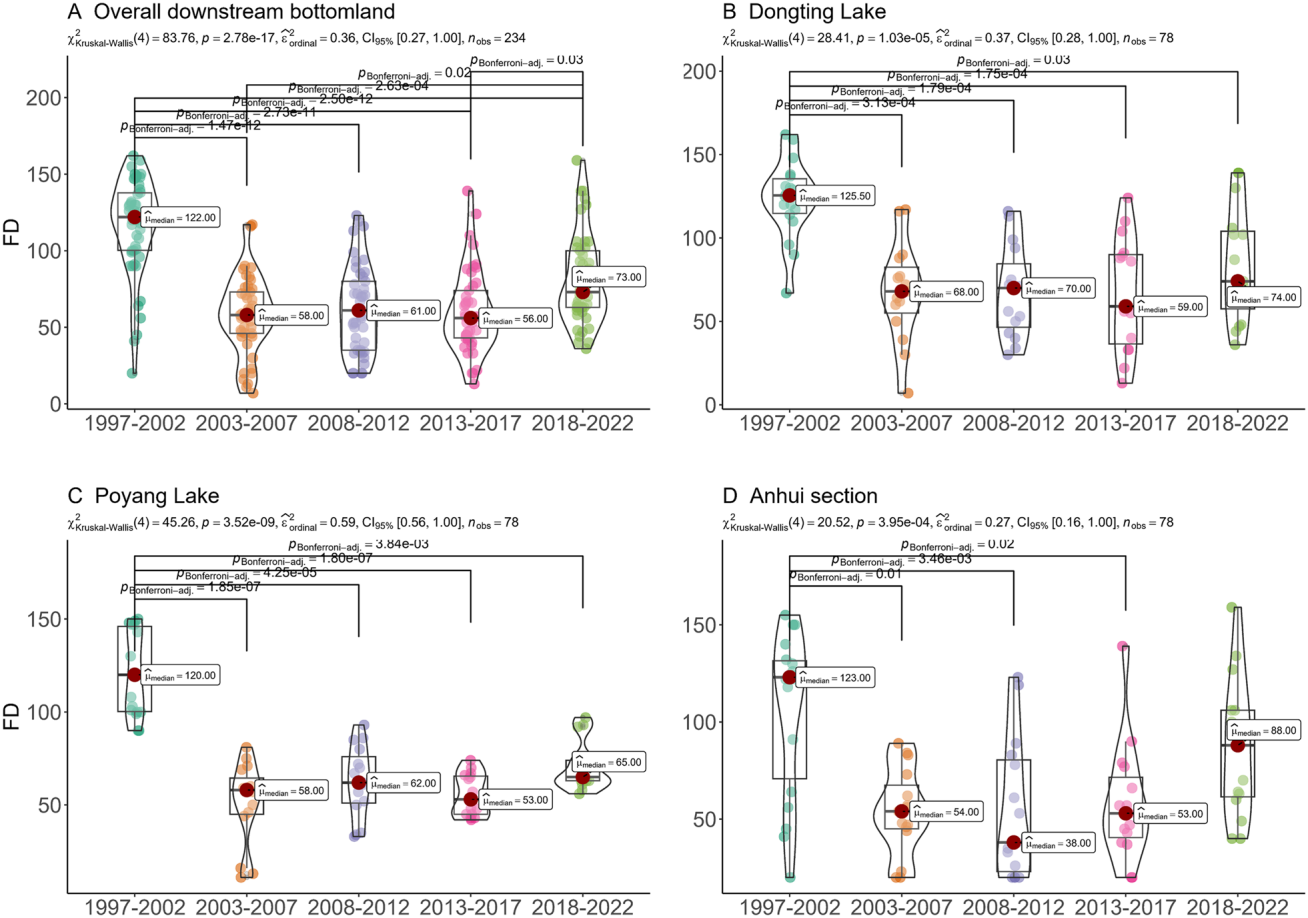
Variation in flood duration from 1997 to 2022: **A** overall bottomlands downstream from the dam; **B**–**D** bottomlands in the Dongting Lake area, Poyang Lake area, and Anhui section, respectively

The FD on the bottomlands decreased from 122 days during 1997–2002 to 57 days during 2003–2012 (Fig. 4A) and then rose again to 65.5 days in 2013–2022 (*P* < 0.001). In the Dongting Lake and Poyang Lake regions, flooding duration fluctuated during 2003–2022 (Fig. 4B, C). However, in the Anhui Section far from the TGD, the FD increased steadily between 2008 and 2022, with durations of 38, 53, and 88 days during 2008–2012, 2013–2017, and 2018–2022, showing an upward trend (Fig. 4D).

**Fig. 5:**
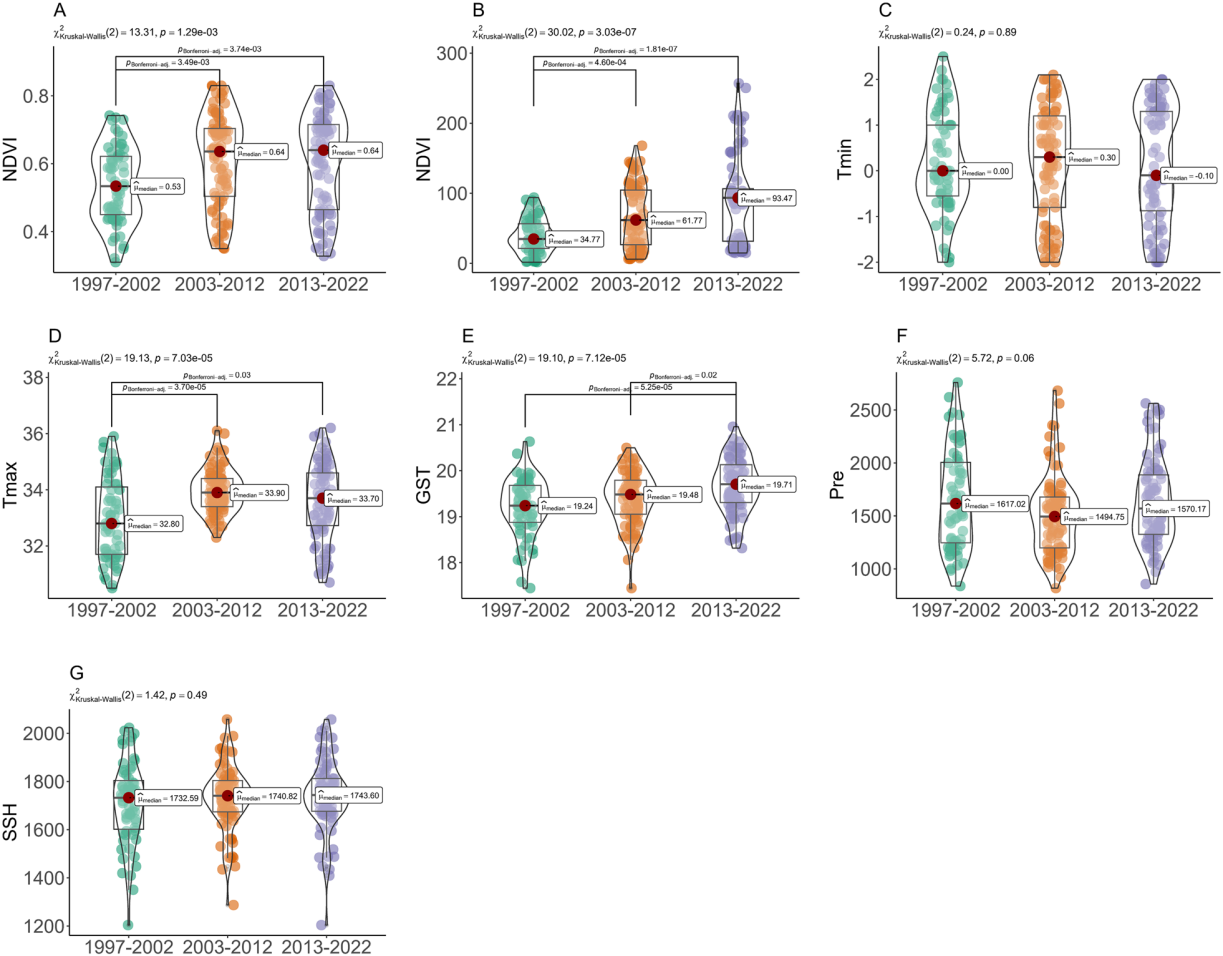
Changes in micro-environmental factors of the snail-breeding bottomland during the periods of 1997–2002, 2003–2012, and 2013–2022, including NDVI (**A**), NL (**B**), Tmin (**C**), Tmax (**D**), GST (**E**), Pre (**F**), and SSH (**G**). NDVI, normalized difference vegetation index; NL, night light index; Tmin, average minimum temperature in January; Tmax, average maximum temperature in July; GST, ground surface temperature; Pre, precipitation; SSH, sunshine hours

As shown in Fig. 5, the bottomland environmental factors experienced various changing trends from 1997 to 2022. The NDVI increased from 0.53 between 1997 and 2002 to 0.64 between 2003 and 2012 and kept steady between 2013 and 2022. Similarly, the NL rose from 34.77 between 1997 and 2002 to 61.82 between 2003 and 2012 and reached 92.89 between 2013 and 2022 (χ^2^ = 30.02,* P* < 0.05). The Tmax showed a rising trend, with values increasing from 32.80 ℃ between 1997 and 2002 to 33.95 ℃ between 2003 and 2012, slightly decreasing to 33.70 ℃ between 2013 and 2022 (χ^2^ = 19.13,* P* < 0.05). The GST exhibited an obvious increasing trend with values of 19.24 ℃, 19.49 ℃, and 19.68 ℃ from 1997 to 2002, 2003 to 2012, and 2013 to 2022 (χ^2^ = 19.10, * P* < 0.05), respectively. However, the differences in the Tmin, precipitation, and SSH between 1997–2002, 2003–2012, and 2013–2022 were not statistically significant.

### Impact of environmental change on snails

**Fig. 6:**
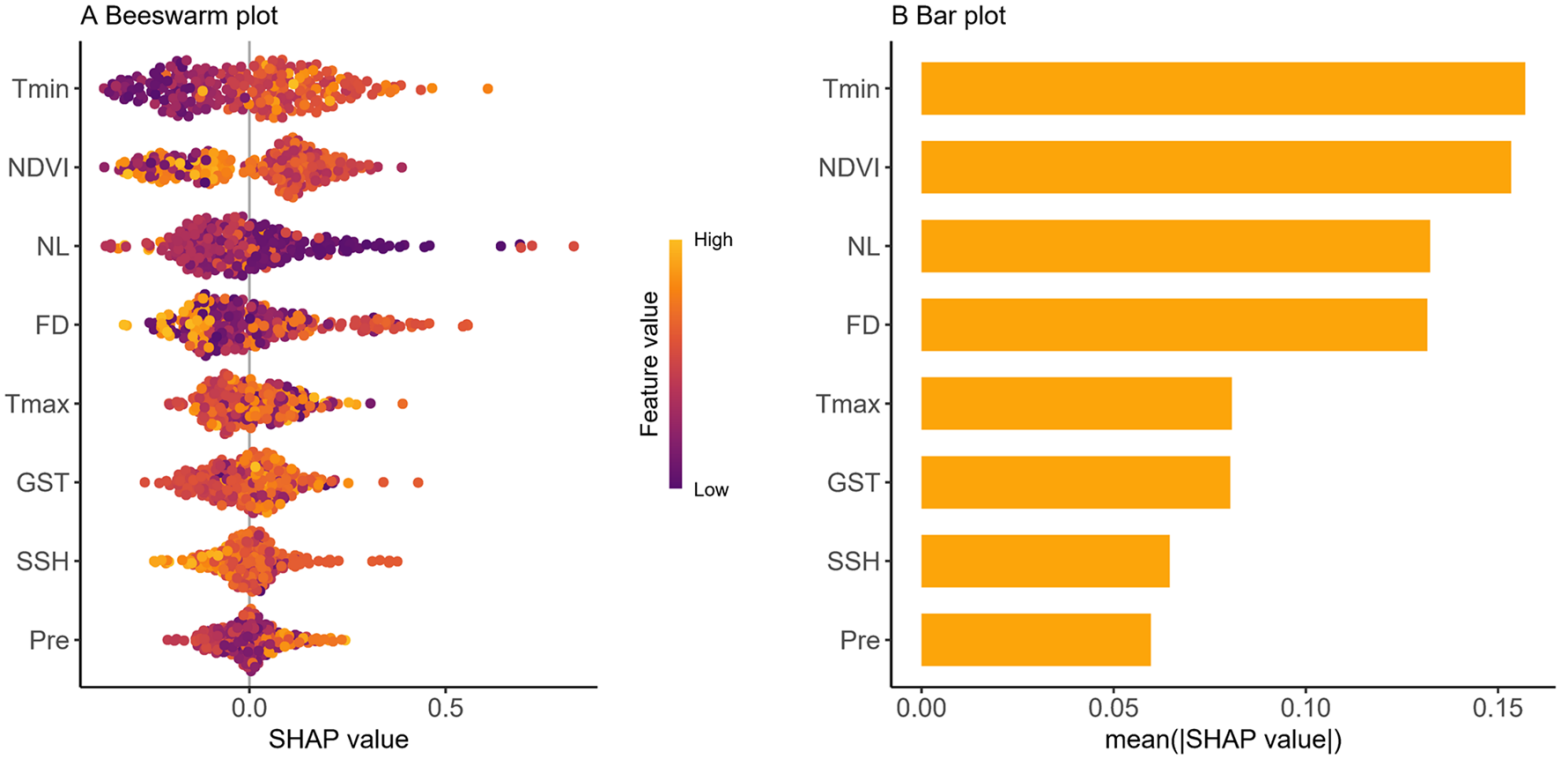
Beeswarm (**A**) and bar (**B**) plot showing the contribution of a single factor affecting snail density. *Tmin* the average minimum temperature in January; *NL* night light index, *FD* flooding duration, *NDVI* normalized difference vegetation index, *Tmax* average maximum temperature in July, *GST* ground surface temperature, *SSH* sunshine hour, *Pre* precipitation

**Fig. 7:**
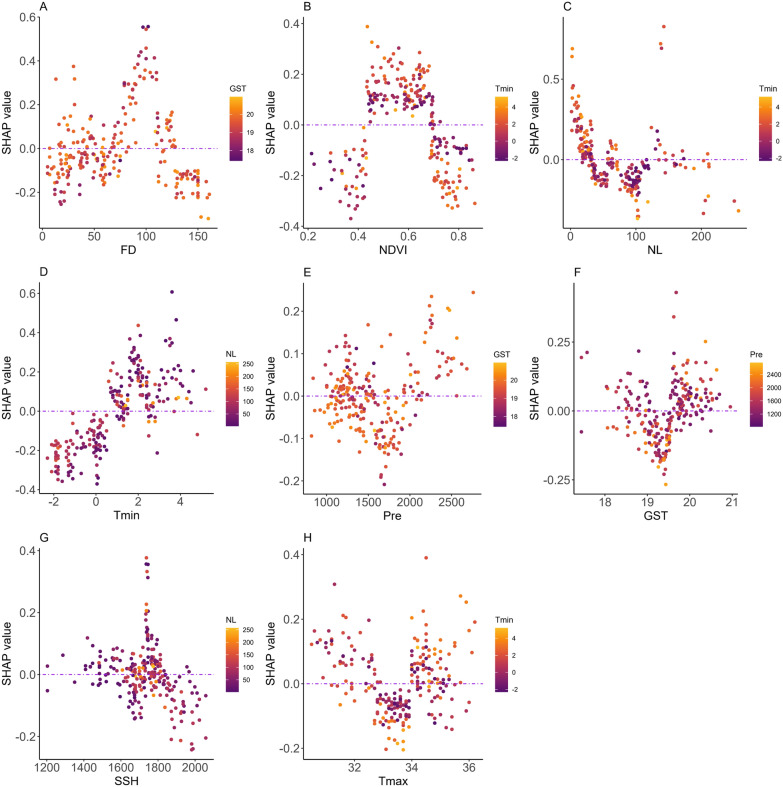
SHAP plot illustrates the relationships between snail density and the factors FD (**A**), NDVI (**B**), NL (**C**), Tmin (**D**), Pre (**E**), GST (**F**), SSH (**G**), and Tmax (**H**). *FD* flooding duration, *NDVI* normalized difference vegetation index, *NL* night light index, *Tmin* the average minimum temperature in January, *Pre* precipitation, *GST* ground surface temperature, *SSH* sunshine hour, *Tmax* average maximum temperature in July

The Tmin, NL (a high night light value reflects the high development and utilization of the bottomland), FD, and NDVI were the four most important factors affecting snail density (Fig. 6). Based on the LightGBM model, a SHAP diagram was conducted to assess the relationship between individual factors and snail density (Fig. 7). At an FD of 50–120 days and NDVI of 0.45 and 0.70, the SHAP values were generally positive, indicating conditions favorable for snail reproduction. Conversely, at a Tmin < 0.3 ℃ and NL > 40, the SHAP values were generally negative, indicating unfavorable conditions for snail breeding. Furthermore, conditions such as higher rainfall and ground surface temperature, and a sunshine duration of 1700 to 1800 h were conducive to snail breeding.

Next, we fitted a total of 24 models including different variables and different nonlinear approximated terms for the environmental factors (Table 1). Model 1 to model 4 included the results of the univariate models and model 5 to model 24 included the results of the multivariate models. Model 20 was selected as the final reported model because it had the lowest DIC value.

**Table 1:** Variable selection of Bayesian spatial-temporal modeling

Model	Included variables	DIC
Model 1	S(FD)^a^	1695.87
Model 2	S(NL)	1765.41
Model 3	S(NDVI)	1763.95
Model 4	S(Tmin)	1765.32
Model 5	S(SSH)	1965.28
Model 6	S(FD) + S(NL)	1543.46
Model 7	S(FD) + S(NDVI)	1547.52
Model 8	S(FD) + S(Tmin)	1678.23
Model 9	S(FD) + S(SSH)	1763.25
Model 10	S(FD) + S(NL) + S(NDVI)	1476.87
Model 11	S(FD) + S(NL) + S(Tmin)	1487.54
Model 12	S(FD) + S(NL) + S(SSH)	1524.62
Model 13	S(FD) + S(NL) + S(NDVI) + S(SSH)	1472.38
Model 14	S(FD) + S(NL) + S(NDVI) + S(Tmin)	1423.82
Model 15	S(FD) + S(NL) + S(NDVI) + S(Tmin) + S(SSH)	1459.73
Model 16	S(FD) + S(NL) + S(NDVI) + S(Tmin) + Tmax	1425.61
Model 17	S(FD) + S(NL) + S(NDVI) + S(Tmin) + Pre	1417.26
Model 18	S(FD) + S(NL) + S(NDVI) + S(Tmin) + GST	1427.26
Model 19	S(FD) + S(NL) + S(NDVI) + S(Tmin) + Pre + S(SSH)	1359.39
Model 20	S(FD) + S(NL) + S(NDVI) + S(Tmin) + Pre + GST	1343.68
Model 21	S(FD) + S(NL) + S(NDVI) + S(Tmin) + Pre + Tmax	1392.47
Model 22	S(FD) + S(NL) + S(NDVI) + S(Tmin) + Pre + GST + Tmax	1349.25
Model 23	S(FD) + S(NL) + S(NDVI) + S(Tmin) + Pre + GST + S(SSH)	1355.37
Model 24	S(FD) + S(NL) + S(NDVI) + S(Tmin) + Pre + GST + Tmax + S(SSH)	1358.57

*FD* flooding duration, *NL* night light index, *NDVI* normalized difference vegetation index, *Tmin* average minimum temperature in January, *SSH* sunshine hour, *Tmax* average maximum temperature in July, *Pre*, precipitation, *GST* ground surface temperature

_a_ S(*variable*) denotes that the variable is included in the form of the smooth spline function

**Fig. 8:**
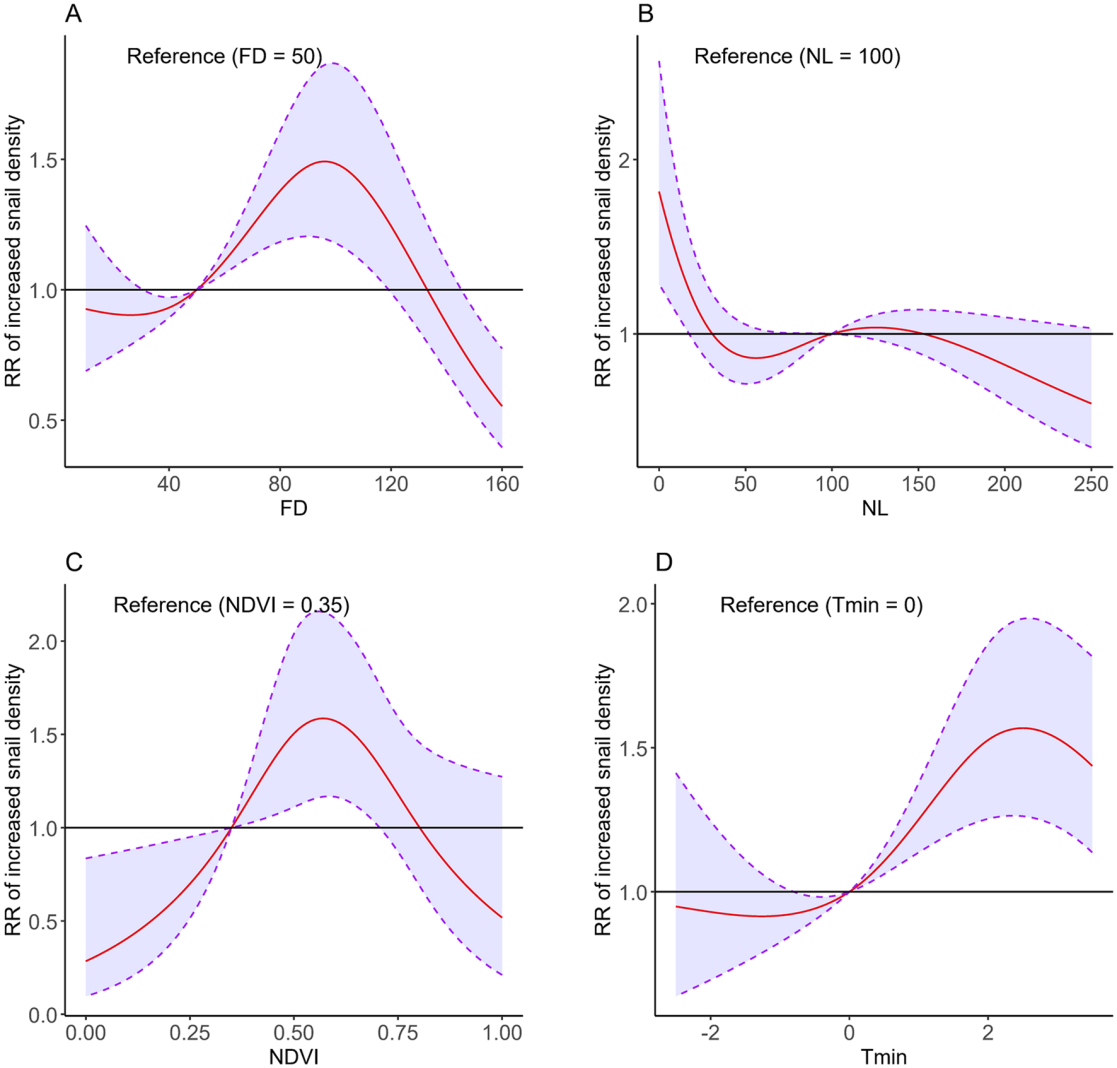
RR of increased snail density associated with FD (**A**), NL (**B**), NDVI (**C**), and Tmin (**D**) in the posterior distribution after adjusting for related factors. *RR* relative risk, *FD* flooding duration, *NL* night light index, *NDVI* normalized difference vegetation index, *Tmin* average minimum temperature in January

The fixed effects results of model 20 are listed in Fig. 8 and Table 2. After adjusting for related confounding factors and the space-time effect (Fig. 8), the RR of increased snail density rose with a flooding duration between 20 to 100 days. However, it decreased as FD increased over 100 days. The relationship between night lighting and snail density was “L” shaped. At low night lighting levels, the RR of increased snail density was higher. As the NDVI increased from 0 to 0.6, the RR of increased snail density also rose. However, the RR then decreased as the NDVI continued to increase. When the Tmin ranged from − 1 to 2 °C, the RR of increased snail density grew as it increased. An increasing trend was detected among bottomlands with a greater GST compared with bottomlands in the first quartile cluster; bottomlands in the second, third, fourth quartile clusters of the GST had higher RR values for snail density, which were 1.271 (95%*CI* 1.082–1.493), 1.302 (95%*CI* 1.146–1.480), and 1.278 (1.048, 1.559), respectively.

**Table 2:** Mean RR of the posterior distribution of ground surface temperature and annual precipitation with increased snail density

Related factors	Unadjusted	Adjusted
	RR (95% CI)	RR (95% CI)
1st quartile of GST	1 (reference)	1 (reference)
2nd quartile of GST	1.278 (0.987, 1.656)	1.271 (1.082, 1.493)^*^
3rd quartile of GST	1.333 (1.021, 1.741)^*^	1.302 (1.146, 1.480)^*^
4th quartile of GST	1.380 (0.990, 1.923)	1.278 (1.048, 1.559)^*^
1st quartile of Pre	1 (reference)	1 (reference)
2nd quartile of Pre	1.142 (0.936, 1.393)	1.125 (0.956, 1.324)
3rd quartile of Pre	1.156 (1.043, 1.282)	1.137 (0.969, 1.207)
4th quartile of Pre	1.201 (1.045, 1.380)	1.177 (0.958, 1.446)

*GST* ground surface temperature, *Pre* precipitation

^*^RR of increased snail density was significantly different at *P* < 0.05

## Discussion

Compared to previous studies [15, 16] conducted during the first decade of the TGD construction, this research revealed that during the second decade of the dam’s operation, various water level indicators (average water level and annual water level fluctuation range) showed an increasing trend (Fig. 3). These changes can be attributed to two primary factors: the increase in extreme rainfall and the varying impacts of the dam's operational phases on downstream water and sediment dynamics. On the one hand, the intensity and frequency of heavy rainstorms in the Yangtze River basin increased under the influence of global climate change [38, 39], contributing to rising water levels. On the other hand, some studies have shown that in the early stages of the TGD, discharge of clean water caused strong scouring of the downstream river channel and promoted decreasing water levels [40, 41]. However, as time went on, the sediment content in the river increased and sediment siltation in the river may have occurred, resulting in a rising water level and establishing a new water-sediment balance [40, 41]. The new water-sediment balance and the change pattern in precipitation might explain why the water level rebounded in 2013–2022 compared with 2003–2012. The recovery of water levels during this period showed regional differences, with a significant rise observed in the Anhui section of the Yangtze River (Fig. 3). Spatially, areas closer to the dam experienced stronger riverbed scouring, while regions farther downstream transitioned more easily to a sediment deposition phase and a rise in water levels [40, 41]. In addition, Guo et al. [42] showed that the most significant impact of the TGD was concentrated on the river section near the TGD, and the degree of impact was five times that of downstream section such as the Anhui section. Therefore, the water levels in the Anhui section are influenced by local tributaries and precipitation more than Dongting Lake. These reasons might be why water level recovery is more obvious in these regions.

The Bayesian spatial-temporal model showed that after adjusting for confounding factors (temperature, vegetation, rainfall, etc.), the RR of increased snail density rose with flooding duration between of 20 and 100 days, while it decreased when FD increased to > 100 days. The continuous change in water level as a fundamental element affected the abundance and distribution of snails. After the construction of the TGD, the water level gradually decreased, and the duration of flooding on the bottomlands was much shorter, affecting the water environment required for normal hatching of snail eggs and snail development [43, 44]. After 2003, the decline in water levels first impacted high-elevation bottomlands, rendering them unsuitable for snail breeding. This was followed by the medium-elevation bottomlands, aligning with the observed decrease in snail density (which began in 2003 for high-elevation areas and in 2004 for medium-elevation areas). Interestingly, snail density in low-elevation bottomlands increased from 2003 to 2005 but then sharply declined starting in 2006. This pattern may be attributed to the prolonged submersion under water of low-elevation bottomlands (e.g. above 100 days), which exceeded the optimal conditions for snail breeding. Initially, the reduction in water levels and shorter flooding duration promoted an increase in snail density. However, as water levels continued to drop beyond the snails' tolerance, their density began to decrease. In addition, the night light index, which reflects the influence of human activities, infrastructure, and other factors on the environment [45], steadily increased in areas surrounding the bottomlands following the construction of the TGD. The study revealed that snail density showed an “L” shape with NL. The RR of increased snail density was lower at higher NL. Since the TGD filled with water, the lower water levels extended the time in which bottomlands remained unflooded. These changes facilitated activities such as farming, cultivation, and tourism and spurred infrastructure development in surrounding areas [46]. Utilization of bottomland resources also contributed to the decline in snail density.

Hydrological changes are not the sole factor influencing *Oncomelania* snail density; micro-environmental conditions, including vegetation and surface temperature, also contribute to the dynamic fluctuations in snail populations. This study revealed that snail density showed a slight rebound, and over half (six/nine) of the bottomlands witnessed a fluctuating increment in snail density between 2013 and 2022. Zhou et al. [47] observed the effects of summer flooding on snail reproduction and found that the number of snail offspring in the 3-month flooding group was 2.5 times that of the non-flooding group. The recovery of the water level facilitated the extension of the flooding duration of the snail habitat, improving the condition of snail breeding and promoting juvenile snails to develop into adult snails [44, 46]. In addition, the ground surface temperature of the bottomland exhibited a rising trend from 1997 to 2022, and an increasing trend of snail density was detected among bottomlands with a greater GST. Laboratory research showed that the development rate of snail eggs could be accelerated with increasing temperature [48, 49]. Previous studies [48, 49] also have shown that extremely high temperatures may limit normal activities of snails such as opening their shells and foraging. However, suitable vegetation could counteract the limiting effect of high temperatures on snails [21, 50]. The NDVI of the bottomland has a small increase within the suitable range for snail survival, which can regulate the extremely high temperature of snail habitats and protect snails from strong sunlight. Therefore, improving hydrological conditions of snail habitats in conjunction with the changing microenvironment contributed to the recovery of snail density. There is also concern in other regions (such as the Philippines) that environmental changes related to dam construction may affect the prevalence of schistosomiasis by affecting freshwater snails [51, 52]. Hence, constructing a real-time monitoring and response system (involving key environmental factors and snails) is crucial for controlling potential risks of schistosomiasis transmission.

This study has some limitations. First, our data came from multiple sources such as the meteorological bureau, hydrological stations, and remote sensing inversion, and there may be measurement bias. Second, studies on the changes in microenvironment, such as vegetation, temperature, etc., need to consider the impact of climate change. In the future, advanced monitoring equipment (such as temperature and humidity monitors, soil sensors, artificial video, etc.) can be installed in snail-breeding bottomlands to precisely collect data covering multiple dimensions of environmental changes in real time. Using big data and artificial intelligence technology, the collected data can be deeply analyzed to predict the reproduction and spreading trends of snails. When the monitoring system detects that environmental changes may cause a surge of snails, an emergency plan should be immediately activated and corresponding control measures should be taken, such as environmental modification, chemical drug release, or biological control. This can not only improve early warning capabilities but also provide a scientific basis for formulating prevention and control measures.

## Conclusions

After construction of the dam, the reduction of the water level and flooding duration of the bottomlands and the increase in the utilization of the bottomland were conducive to reducing snail density. However, over time, the inhibitory effect of the TGD on the snail may be weakening, which is mainly manifested in the rising and unstable water level trend, especially in areas far from the TGD. Environmental changes in the snail-breeding bottomland may bring uncertainty to the snail population. In recent years, the rebound of the snail density in local bottomlands may be related to the rise in water levels and the change in the microenvironment. Establishing an efficient monitoring and response system is crucial for the precise control of snails and the elimination of schistosomiasis.

## Supplementary Information

Additional file 1: Table S1. Information on the nine bottomlands. Table S2. Correlation analysis of variables. Table S3. Results of Shapiro-Wilk test. Table S4. Characteristics of variables in the bottomlands in the middle and low reaches of the Yangtze River basin.

## Data Availability

Supporting data for the conclusions of this article are included within the article. The raw data supporting the conclusions of this article will be made available upon reasonable request.

## Abbreviations

TGD: The Three Gorges Dam
FD: Flooding duration
NDVI: Normalized difference vegetation index
NL: Nighttime light index
SSH: Sunshine hour
GST: Ground surface temperature
Pre: Precipitation
Tem: Temperature
RH: Relative humidity
Tmin: Average minimum temperature in January
Tmax: Average maximum temperature in July
RR: Relative risk
AI: Artificial intelligence
SHAP: Shapley additive explanations
LightGBM: Light gradient boosting machine
DIC: Deviance information criterion
