# Supplementary material for: Decoding the impact of environmental shifts on snail density dynamics in the Yangtze River basin: a 26-year study
Source: Parasit Vectors. 2025 Apr 26;18:156. doi: 10.1186/s13071-025-06782-3 (PMC12032699; doi:10.1186/s13071-025-06782-3)
Supplement: Supplementary file 1 — Additional file 1: Table S1. Information on the nine bottomlands. Table S2. Correlation analysis of variables. Table S3. Results of Shapiro-Wilk test. Table S4. Characteristics of variables in the bottomlands in the middle and low reaches of the Yangtze River basin. [file 13071_2025_6782_MOESM1_ESM.docx]

Supplementary materials

Table S1 The information of 9 bottomlands

| Region | Bottomland name | Elevation category | longitude | latitude |
| --- | --- | --- | --- | --- |
| Dongting Lake | Matian | High-elevation | E112.1270° | N29.1978° |
|  | Xingang | Middle-elevation | E113.3189° | N29.6146° |
|  | Junshan Park | Low-elevation | E112.9963° | N29.3507° |
| Poyang Lake | Ganyu | High-elevation | E116.2585° | N28.9049° |
|  | Fanlong | Middle-elevation | E116.1222° | N29.7213° |
|  | Chenjiamen | Low-elevation | E116.0843° | N29.3614° |
| Anhui Section | Xin | High-elevation | E118.3383° | N31.4913° |
|  | Lao | Middle-elevation | E117.1971° | N30.4994° |
|  | Chenqiao | Low-elevation | E117.7605° | N30.9506° |

Table S2 Correlation analysis of variables

|  | NDVI | NL | SSH | RH | FD | Tmin | Tmax | Tem | Pre | GST |
| --- | --- | --- | --- | --- | --- | --- | --- | --- | --- | --- |
| NDVI | 1 |  |  |  |  |  |  |  |  |  |
| NL | 0.219* | 1 |  |  |  |  |  |  |  |  |
| SSH | -0.142* | 0.27* | 1 |  |  |  |  |  |  |  |
| RH | -0.154* | -0.077 | -0.088 | 1 |  |  |  |  |  |  |
| FD | -0.375* | -0.076 | 0.031 | 0.763* | 1 |  |  |  |  |  |
| Tmin | -0.019 | -0.209* | -0.086 | 0.303* | 0.082 | 1 |  |  |  |  |
| Tmax | 0.093 | 0.148* | 0.047 | -0.252* | -0.063 | 0.088 | 1 |  |  |  |
| Tem | -0.178* | -0.125 | -0.152* | -0.002 | 0.188* | 0.163* | 0.215* | 1 |  |  |
| Pre | -0.124 | 0.051 | -0.238* | 0.438* | 0.234* | 0.144* | -0.115 | 0.192* | 1 |  |
| GST | 0.142* | 0.185* | 0.072 | -0.095 | 0.055 | 0.118 | 0.285* | 0.815* | -0.093 | 1 |

Note: NDVI normalized difference vegetation index; NL annual night-time light index; SSH sunshine hour; RH average annual relative humidity; FD flood duration; Tmin average minimum temperature in Jan; Tmax average maximum temperature in Jul; Tem annual average temperature; Pre total precipitation; GST average annual ground surface temperature.

* The correlation between two variables was statistically significant at *P* < 0.05

Table S3 The results of Shapiro-Wilk test

| Variable name | W | P-value |
| --- | --- | --- |
| FD | 0.9796 | 1.84E-03 |
| NDVI | 0.9625 | 8.04E-06 |
| NL | 0.9102 | 1.18E-10 |
| GST | 0.9863 | 2.41E-02 |
| Tmax | 0.9801 | 2.21E-03 |
| Tmin | 0.9530 | 6.70E-07 |
| Tem | 0.9387 | 2.51E-08 |
| Pre | 0.9606 | 4.77E-06 |
| SSH | 0.9691 | 5.63E-05 |
| RH | 0.9720 | 1.38E-04 |

Note: FD flood duration; NDVI normalized difference vegetation index; NL annual night-time light index; GST average annual ground surface temperature; Tmax average maximum temperature in Jul; Tmin average minimum temperature in Jan; Tem annual average temperature; Pre total precipitation; SSH sunshine hour; RH average annual relative humidity.

Table S4 The characteristics of variables in the bottomlands in the middle and low reaches of Yangtze River basin

| Variable name (unit) | Mean | SD | Min | P25 | Median | P75 | Max. |
| --- | --- | --- | --- | --- | --- | --- | --- |
| FD (day) | 73.56 | 44.31 | 5 | 36 | 68.5 | 112 | 161 |
| High water level (m) | 21.51 | 9.65 | 8.27 | 14.52 | 17.49 | 31.6 | 41.8 |
| Low water level (m) | 11.22 | 8.97 | 1.4 | 5.24 | 6.37 | 20.13 | 29.45 |
| Variation amplitude (m) | 10.29 | 2.55 | 4.67 | 8.65 | 10.3 | 11.85 | 16.73 |
| NDVI | 0.59 | 0.14 | 0.31 | 0.46 | 0.61 | 0.7 | 0.83 |
| NL | 69.31 | 52.62 | 1.65 | 25.16 | 57.79 | 100.32 | 256.83 |
| SSD (hour) | 1730 | 147.2 | 1204 | 1665 | 1740 | 1808 | 2058 |
| RH (1%) | 76.25 | 3.18 | 67.54 | 73.95 | 76.86 | 78.62 | 84.38 |
| Tmin (℃) | 0.99 | 1.74 | -2.3 | -0.1 | 1.1 | 2.2 | 5.2 |
| Tmax (℃) | 33.56 | 1.25 | 30.5 | 32.8 | 33.7 | 34.5 | 36.2 |
| Tem (℃) | 17.65 | 0.74 | 15.88 | 17.1 | 17.63 | 18.15 | 19.59 |
| GST (℃) | 19.45 | 0.62 | 17.45 | 19.05 | 19.5 | 19.85 | 20.96 |
| Pre (mm) | 1562.9 | 421.71 | 818.5 | 1242.1 | 1474.1 | 1804.1 | 2759.8 |

Note: FD flood duration; NDVI normalized difference vegetation index; NL annual night-time light index; GST average annual ground surface temperature; Tmax average maximum temperature in Jul; Tmin average minimum temperature in Jan; Tem annual average temperature; Pre total precipitation; SSH sunshine hour; RH average annual relative humidity.
